# Novel diagnostic biomarkers related to immune infiltration in Parkinson’s disease by bioinformatics analysis

**DOI:** 10.3389/fnins.2023.1083928

**Published:** 2023-01-26

**Authors:** Pengfei Zhang, Liwen Zhao, Hongbin Li, Jie Shen, Hui Li, Yongguo Xing

**Affiliations:** ^1^Department of Neurosurgery, Beichen Traditional Chinese Medical Hospital Tianjin, Tianjin, China; ^2^Department of Neurosurgery, Tianjin Medical University General Hospital Airport Site, Tianjin, China; ^3^Department of Neurology, Beichen Traditional Chinese Medical Hospital Tianjin, Tianjin, China

**Keywords:** Parkinson’s disease, bioinformatics analysis, immune infiltration, weighted gene co-expression network analysis (WGCNA), hub genes, gene set enrichment analysis

## Abstract

**Background:**

Parkinson’s disease (PD) is Pengfei Zhang Liwen Zhao Pengfei Zhang Liwen Zhao a common neurological disorder involving a complex relationship with immune infiltration. Therefore, we aimed to explore PD immune infiltration patterns and identify novel immune-related diagnostic biomarkers.

**Materials and methods:**

Three substantia nigra expression microarray datasets were integrated with elimination of batch effects. Differentially expressed genes (DEGs) were screened using the “limma” package, and functional enrichment was analyzed. Weighted gene co-expression network analysis (WGCNA) was performed to explore the key module most significantly associated with PD; the intersection of DEGs and the key module in WGCNA were considered common genes (CGs). The CG protein–protein interaction (PPI) network was constructed to identify candidate hub genes by cytoscape. Candidate hub genes were verified by another two datasets. Receiver operating characteristic curve analysis was used to evaluate the hub gene diagnostic ability, with further gene set enrichment analysis (GSEA). The immune infiltration level was evaluated by ssGSEA and CIBERSORT methods. Spearman correlation analysis was used to evaluate the hub genes association with immune cells. Finally, a nomogram model and microRNA-TF-mRNA network were constructed based on immune-related biomarkers.

**Results:**

A total of 263 CGs were identified by the intersection of 319 DEGs and 1539 genes in the key turquoise module. Eleven candidate hub genes were screened by the R package “UpSet.” We verified the candidate hub genes based on two validation sets and identified six (SYT1, NEFM, NEFL, SNAP25, GAP43, and GRIA1) that distinguish the PD group from healthy controls. Both CIBERSORT and ssGSEA revealed a significantly increased proportion of neutrophils in the PD group. Correlation between immune cells and hub genes showed SYT1, NEFM, GAP43, and GRIA1 to be significantly related to immune cells. Moreover, the microRNA-TFs-mRNA network revealed that the microRNA-92a family targets all four immune-related genes in PD pathogenesis. Finally, a nomogram exhibited a reliable capability of predicting PD based on the four immune-related genes (AUC = 0.905).

**Conclusion:**

By affecting immune infiltration, SYT1, NEFM, GAP43, and GRIA1, which are regulated by the microRNA-92a family, were identified as diagnostic biomarkers of PD. The correlation of these four genes with neutrophils and the microRNA-92a family in PD needs further investigation.

## Introduction

Neurological disorders, as the main cause of disability worldwide, impose a major financial burden (Feigin et al., 2019). Compared to other neurological disorders, Parkinson’s disease (PD) has shown the fastest growth in prevalence and incidence in recent years (Bloem et al., 2021). The main characteristic features of PD include progressive loss of neurons in specific areas of the substantia nigra and the presence of Lewy bodies in the brain (Wakabayashi et al., 2013), which lead to dysfunction in patients. There have been significant advances in the treatment of PD, such as dopamine substitution and deep-brain stimulation, which retard symptom progression and improve quality of life for decades after disease onset (LeWitt and Fahn, 2016; Limousin and Foltynie, 2019). However, PD eventually leads to severe disability, which remains a healthcare challenge. Therefore, modifying PD progression and delaying disability are still key problems that need to be solved. When early clinical signs are inadequate for diagnosis of PD, in particular a lack of typical motor symptoms, diagnosis is often delayed and misdiagnosis may occur (Feigin et al., 2019; Bloem et al., 2021). By the time a diagnosis of PD is made, a substantial proportion of neurons in the brain have been lost (Gaenslen et al., 2011; Postuma and Berg, 2016). Thus, new diagnostic methods, including biomarkers that can identify individuals at risk and early before clinical manifestation of the onset of motor symptoms are needed.

In recent years, growing evidence has indicated that the immune system is involved in the pathophysiology of PD and increases the progression of PD (Öberg et al., 2021; Zhang et al., 2021). For instance, immune infiltrating cells CD8+ and CD4+ T cells, which were significantly different in PD samples compared with control animal models (Brochard et al., 2008; Harms et al., 2017), were related to dopaminergic neuron cell loss in the PD group (Brochard et al., 2008; Williams et al., 2021). Notably, a high proportion of substantia nigra CD8 T-cell infiltration has been considered an early alteration in PD, even occurring before death of dopamine neuronal cells and α-synuclein aggregation, which is also associated with progression of PD (Galiano-Landeira et al., 2020). However, the pathological mechanism underlying immune infiltration in PD lacks comprehensive evidence. Thus, understanding the mechanism of immune regulation in PD and identifying reliable biomarkers related to immune regulation can guide clinical diagnosis and immune strategies for treatment of the disease.

MicroRNA (miRNA), a small single-stranded non-coding RNA molecule, binds to mRNA and induces mRNA degradation and translational repression for posttranscriptional regulation of gene expression. Recent studies have elucidated that dysregulated expression of miRNAs plays a substantial role in regulating PD (Briggs et al., 2015; Nair and Ge, 2016). For instance, miR-153 can significantly reduce expression of synuclein-alpha (SNCA) (Je and Kim, 2017), which has been confirmed to be relevant to the pathogenesis of PD (Lesage et al., 2020; Kung et al., 2022). Moreover, miRNAs are not only related to dopaminergic neuron survival (Kabaria et al., 2015) and neuroinflammation (Yao et al., 2018), but can also serve as diagnostic biomarkers (Shu et al., 2020) and therapeutic tools for PD (Gan et al., 2019; Nies et al., 2021). Therefore, miRNAs can provide useful insight into the pathophysiology of PD to identify new therapeutic targets and strategies to slow or reverse neurodegeneration.

In recent years, with the development of microarray technology, bioinformatics analysis has been widely applied to identify potential novel biomarkers and reveal key pathways to explore the pathogenesis and drug targets of different diseases (Zhou et al., 2021). In this study, we conducted systematic bioinformatics analysis to identify novel immune infiltration-related diagnosis genes and understand the potential immune mechanism during the development of PD.

We integrated three datasets from the Gene Expression Omnibus (GEO) database, including 38 substantia nigra samples from the PD group and 29 normal samples. Differential expression gene (DEG) analysis of the integrated dataset comparing PD samples with normal controls, Gene Ontology (GO) functional analysis, Kyoto Encyclopedia of Genes and Genomes (KEGG) pathway analysis, weighted gene co-expression network analysis (WGCNA), and protein–protein interaction (PPI) analysis were successively performed. Next, six hub genes were identified after validation using another two cohorts. The diagnostic effectiveness of the hub genes, gene set enrichment analysis (GSEA) of the hub genes, and the correlation between the hub genes and immune infiltration type were investigated. Then, four immune infiltration-related marker genes were confirmed. Finally, a nomogram model and miRNA-TF-mRNA network were constructed based on four immune infiltration-related marker genes, constituting potential biomarkers for the early diagnosis and treatment of PD.

## Materials and methods

### Data collection and preprocessing

We used the keyword ‘‘Parkinson’s disease’’ to search the GEO^1^ (Clough and Barrett, 2016). Five datasets were downloaded, and those datasets met of the following inclusion criteria: (1) related to *Homo sapiens*; (2) datasets containing Parkinson’s patients and control subjects; and (3) tissue derived from the substantia nigra.

We downloaded the original data files (*.CEL) of the four datasets (GSE8397, GSE20163, GSE20164, and GSE20292), which were all based on the GPL96 (HG-U133A) Affymetrix Human Genome U133A Array (Affymetrix, Santa Clara, CA, United States). The GSE26927 dataset, including 12 PD substantia nigra tissue samples and 8 normal substantia nigra samples, was based on the GPL6255 platform Illumina humanRef-8 v2.0 expression beadchip (Illumina Inc., Bethesda, MD, United States). The GSE8397 dataset (GPL96, HG-U133 A chips) consisted of 47 individual brain tissue samples, including substantia nigra tissues of 24 patients with PD and 15 control samples. GSE20163 contained 17 substantia nigra tissue samples, including 9 patients with PD and 8 normal substantia nigra tissue samples. GSE20164 consisted of 11 substantia nigra tissue samples, including 5 PD samples and 6 normal samples. GSE20292 consisted of 29 substantia nigra tissue samples, including 11 PD samples and 18 normal samples. GSE26927 was used to validate the hub genes. The raw data files (*.CEL) of the four datasets (GSE8397, GSE20163, GSE20164, and GSE20292) were processed by the “affy” package (version 1.74.0) (Gautier et al., 2004). The robust multichip average (RMA) method was used for the above four datasets to obtain the gene expression matrix after background correction, normalization and calculation of expression values in the “affy” package.

**FIGURE 1:**
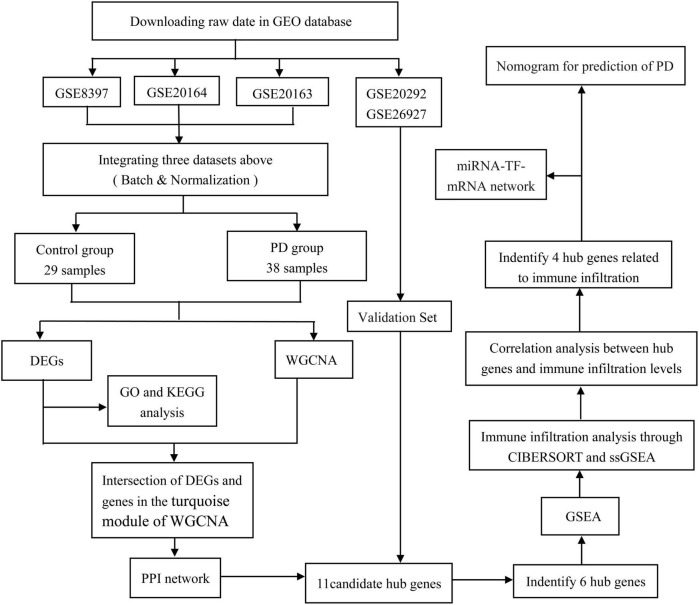
Flowchart of the study. GEO, gene expression omnibus; DEGs, differentially expressed genes; WGCNA, weighted gene co-expression network analysis; ssGSEA, single-sample gene set enrichment analysis; GSEA, gene set enrichment analysis; GO, gene ontology; KEGG, Kyoto Encyclopedia of Genes and Genomes; PPI, protein–protein interaction; PD, Parkinson’s disease; TF, transcriptional factor.

We used the ‘‘limma’’ package (version 3.52.2) of *R* software^2^ (ver. 4.2.0) (Ritchie et al., 2015) to match the identity document (IDs) of the datasets with that of the gene (gene symbol) based on each platform annotation file; empty probes that did not match the gene symbol were removed. If multiple probes corresponded to the same gene symbol, the maximum expression value was taken as its expression value. The gene expression profiles of GSE8397, GSE20163, and GSE20164 were integrated by the “limma” package in *R*. The combined dataset is processed using the surrogate variable analysis (SVA) package (version 3.44.0) (Leek et al., 2019) to remove batch effects and other unwanted variations in high-throughput experiments. Supplementary Table 1 shows the detailed information of the five datasets, and a flow chart of the study design is shown in Figure 1.

### Analysis of differentially expressed genes (DEGs)

The “limma” package was used to normalize the integrated dataset with the ‘normalize Between Arrays’ function in *R* software (version 4.2.0) and then screen DEGs between 38 patients with PD and 29 normal substantia nigra tissues from control patients with the threshold of adjusted *p*-value < 0.05 and | log2 Fold change (FC)| > 0.5. Heatmap and volcano plots of DEGs were created by the “pheatmap” package (version 1.0.12) and the “ggplot2” package (version 3.3.6) in *R* software (version 4.2.0), respectively.

### Gene ontology (GO) and Kyoto Encyclopedia of Genes and Genomes (KEGG) analyses

We examined GO functions from three classifications, including biological processes (BP), cellular component (CC), and molecular function (MF), to further explore the potential molecular mechanisms of DEGs. KEGG was used to integrate pathway information for those DEGs. The R project was used to perform GO and KEGG analyses based on the “clusterProfiler” (Wu et al., 2021), “org.Hs.e.g.db” (Carlson et al., 2019), “ggplot2” (Villanueva and Chen, 2019), “enrichplot” (Yu, 2019), and “DOSE” (Yu et al., 2015) packages, and adjusted *p* < 0.05 was considered to be statistically significant.

### Construction of weighted gene co-expression network analysis (WGCNA) and identification of hub modules

Weighted gene co-expression network analysis, a systematic biology approach, was utilized to construct a co-expressed gene network and to explore genes closely associated with the clinical phenotype. First, according to variation across samples in the integrated dataset, the top 5000 genes were imported into WGCNA using the “WGCNA” package (version 1.71) (Langfelder and Horvath, 2008). Second, all samples were clustered, and discrete samples were removed to ensure the reliance of the network construction results. Third, the soft threshold parameter was calculated, and the optimal parameter β was selected to form the scale-free network based on the scale independence and mean connectivity. According to the suitable power of β = 7 (*R*^2^ = 0.85), the topological overlap matrix (TOM) and corresponding dissimilarity (1-TOM) were calculated. Fourth, through the dynamics cut tree algorithms, hierarchical clustering genes were identified, and then similar genes were classified into the same modules based on the TOM-based dissimilarity measure.

Each module of the gene dendrogram contained at least 50 genes, and similar modules were merged, with a height cutoff of 0.25. Finally, the module membership (MM) and gene significance (GS) were measured. The relevance between module eigengenes (MEs) and clinical traits was assessed by the Pearson correlation test and was shown with a heatmap to identify the most significant modules associated with MEs. Significant module genes were selected for further analysis.

### Construction of a protein–protein interaction (PPI) network and identification of candidate hub genes

The “VennDiagram” package (Chenn, 2018) (version 1.7.3) was used to obtain intersecting common genes (CGs) between DEGs and the genes in the most significant module of WGCNA. The PPI network of CGs was analyzed with the Search Tool for the Retrieval of Interacting Genes (STRING^3^; version 11.0) online database (Szklarczyk et al., 2019). In addition, we selected PPI interaction pairs with a significance cutoff of interaction score over 0.4 while hiding disconnected nodes in the network. Finally, the results of the PPI network were visualized in Cytoscape (Shannon et al., 2003) (version 3.9.1). We selected the CytoHubba (version 0.1) plugin in Cytoscape by nine algorithms, namely, maximal clique centrality (MCC), maximum neighborhood component (MNC), node connection degree (Degree), edge percolated component (EPC), BottleNeck, closeness, radiality, stress, and betweenness, to detect the top 30 genes by each approach from the PPI network. Then, the number of nodes for each gene (PPICount) was calculated, and the top 30 genes with the largest nodes were obtained in *R* software. For this study, nine approaches of CytoHubba and PPIcount were used to screen candidate hub genes by the “UpSetR” package (Gehlenborg, 2019) (version 1.4.0) in *R*.

### Identification of hub genes and diagnostic implications of hub genes for PD

Expression of candidate hub genes was extracted from GSE20292 and GSE26927. The difference between PD and normal samples of each candidate hub gene was calculated and visualized by the “ggpurb” package (Kassambara, 2020). Candidate hub genes that were statistically significant in both training and validation sets were considered hub genes. A *p*-value of <0.05 was considered significant.

To evaluate the ability of the hub genes in both the training and validation sets to identify PD, the “pROC” package (Robin et al., 2011) was used to conduct receiver operating characteristic (ROC) curve analysis. The area under the curve (AUC) value was used to examine the diagnostic effectiveness in discriminating PD from control samples in both training and validation sets.

### Single-gene gene set enrichment analysis (GSEA)

Through the median expression value of six hub genes (SYT1, GAP43, SNAP25, GRIA1, NEFL, and NEFM), we divided the 38 PD substantia nigra tissues into low- and high-expression groups based on each hub gene. Then, to further explore the function of hub genes, single-gene GSEA was implemented by the ordered gene expression matrix based on the Pearson correlation between each hub gene and other genes in *R* software using the “clusterProfiler” (Wu et al., 2021) and “enrichplot” (Yu, 2019) packages. A *p*-value of <0.05 was considered significant.

### Evaluation of immune cell infiltration by ssGSEA and CIBERSORT

Single-sample gene set enrichment analysis (ssGSEA) was implemented to estimate infiltration levels of 16 immune cells and 13 immune functions on the basis of expression profiling between the PD and control samples using the “GSVA” package (version 1.40.1) (Hänzelmann et al., 2013). The “pheatmap” package (version 1.0.12) (Kolde, 2019) was used to visualize the heatmap of 29 immune cells and immune functions. The correlation heatmap between risk scores and the scores of 16 immune cells and 13 immune functions was generated using the “corrplot” package (Version 0.90) (Wei and Simko, 2021). Boxplots were separately generated using the “ggpubr” package (version 0.4.0) (Kassambara, 2020) and the “reshape2” package (Wickham, 2007). We used Spearman correlation analysis to reveal the correlations between the candidate hub genes and 29 immune cells and immune functions.

In addition, the CIBERSORT algorithm (Newman et al., 2015) was applied to quantify and calculate the proportion of 22 types of infiltrating immune cells among the merged expression profile; CIBERSORT filters the samples at *p* < 0.05. A bar plot was generated to show the percentage of 22 types of immune cells in each sample. The heatmap, violin plot, and correlation heatmap were generated using the “pheatmap” package (Kolde, 2019), “vioplot” package (version 0.3.7) (Adler and Kelly, 2020), and “corrplot” package (Wei and Simko, 2021) by the R program, respectively.

Correlations between the candidate hub genes and 20 types of immune infiltrations were calculated and visualized by using Spearman correlation analysis. Based on ssGSEA and CIBERSORT, four hub genes that were most relevant to immune infiltration were selected.

### Construction of a nomogram model for PD

We established a nomogram model based on four immune-related biomarker genes for predicting the occurrence of PD using the “rms” package (Harrell, 2021). The accuracy of the nomogram was assessed through calibration curve analyses. In addition, the AUC value was utilized to quantify the predictive performance of the nomogram model based on ROC curve analyses using the “ROCR” package (Sing et al., 2005).

### Analysis of transcription factors (TFs) and miRNAs of immune-related hub genes

We searched the target miRNAs of immune-related biomarker genes in the miRWalk (Dweep et al., 2011), RNAInter (Kang et al., 2022), and TargetScan (Agarwal et al., 2015) databases and retained common miRNAs. Moreover, we determined the transcription factors (TFs) for the hub genes in the Enrichr database^4^ and screened the results with a *p*-value < 0.05. Finally, a miRNA-TF-mRNA regulatory network was constructed using Cytoscape.

### Statistical analysis

We used version 4.2.0 of *R* software (limma, ggpurb, pheatmap, violplot, corrplot package, and so on) for all statistical analyses. Student’s *t* test was applied to compare the mean difference between groups. Correlation between variables was determined using Pearson’s or Spearman’s correlation test. Two-tailed *p*-values < 0.05 were considered significant.

## Results

### Identification of DEGs

**FIGURE 2:**
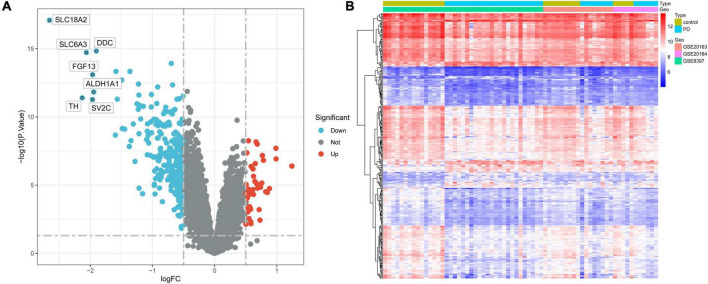
Identification of DEGs in the integrated dataset. **(A)** Volcano plot of all DEGs. The tomato nodes represent upregulated DEGs with *p*-value < 0.05 and logFC > 0.5; the cyan nodes represent downregulated DEGs with *p*-value < 0.05 and logFC < –0.5. **(B)** Heatmap of DEGs in PD samples vs. normal samples. Each row of the heatmap represents one gene, and each column represents one sample. The red and blue colors represent gene expression levels corresponding to upregulated and downregulated expression. DEGs, differentially expressed genes; FC, fold change; PD, Parkinson’s disease.

Differentially expressed genes of the integrated dataset were analyzed using the “limma” package. A total of 319 genes were differentially expressed between 38 PD samples and 29 normal substantia nigra tissue samples, with 45 genes being upregulated and 274 downregulated. The volcano plot and heatmap of DEGs are shown in Figures 2A, B, respectively.

### GO and KEGG analyses of DEGs

**FIGURE 3:**
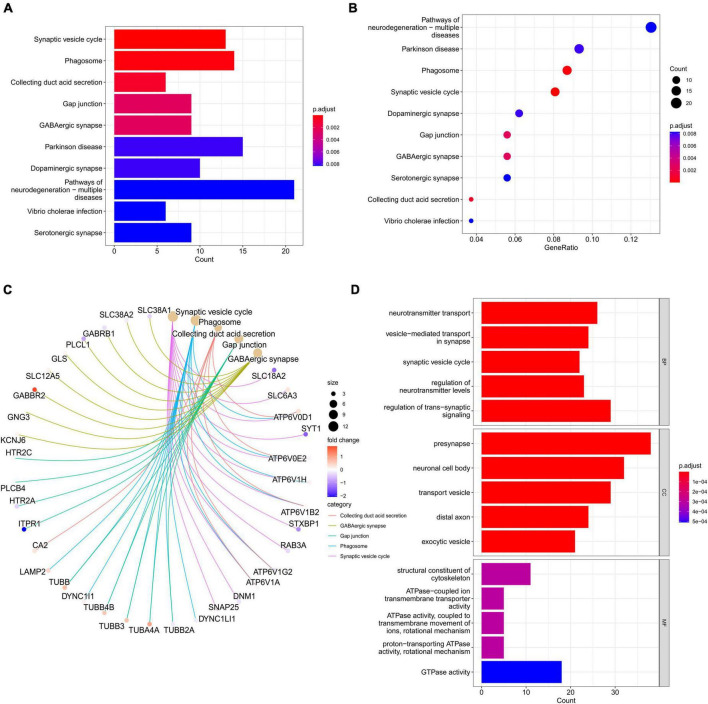
Gene ontology and KEGG pathway enrichment analyses of DEGs. **(A)** Barplot of KEGG analysis based on the obtained 263 genes. **(B)** The bubble diagrams show the top ten significantly enriched terms in KEGG analysis. The *X*-axis is the GeneRatio (gene count/gene size) of the term, and the *Y*-axis denotes the name of the term. The darker the color is, the smaller the adjusted *p*-value is. **(C)** Subnetwork showing the top five KEGG pathways and related genes. **(D)** The top 5 terms for BP, CC, and MF with *p* < 0.05 are shown. DEGs, differentially expressed genes; KEGG, Kyoto Encyclopedia of Genes and Genomes; GO, gene ontology; BP, biological processes; CC, cell component; MF, molecular function.

Differentially expressed genes were assessed through GO and KEGG analyses to explore the biological functions associated with PD. KEGG enrichment analysis results showed that DEGs are mainly related to synaptic vesicle cycle, phagosome, collecting duct acid secretion, gap junction, GABAergic synapse, and Parkinson’s disease, among others (Figures 3A–C and Supplementary Table 2). GO enrichment analysis indicated DEGs to be associated with neurotransmitter transport, vesicle-mediated transport in synapse, synaptic vesicle cycle, regulation of neurotransmitter levels, and regulation of trans-synaptic signaling, among others, in biological processes (BP) analysis. Cellular component (CC) analysis showed the DEGs to be mainly enriched in presynapse, neuronal cell body, transport vesicle, distal axon, and exocytic vesicle, among others. The top five significant terms enriched in molecular function (MF) analysis were structural constituent of cytoskeleton, ATPase-coupled ion transmembrane transporter activity, ATPase activity (coupled to transmembrane movement of ions, rotational mechanism), proton-transporting ATPase activity (rotational mechanism), and GTPase activity (Figure 3D and Supplementary Table 2).

### Construction of a weighted co-expression network and identification of hub modules

**FIGURE 4:**
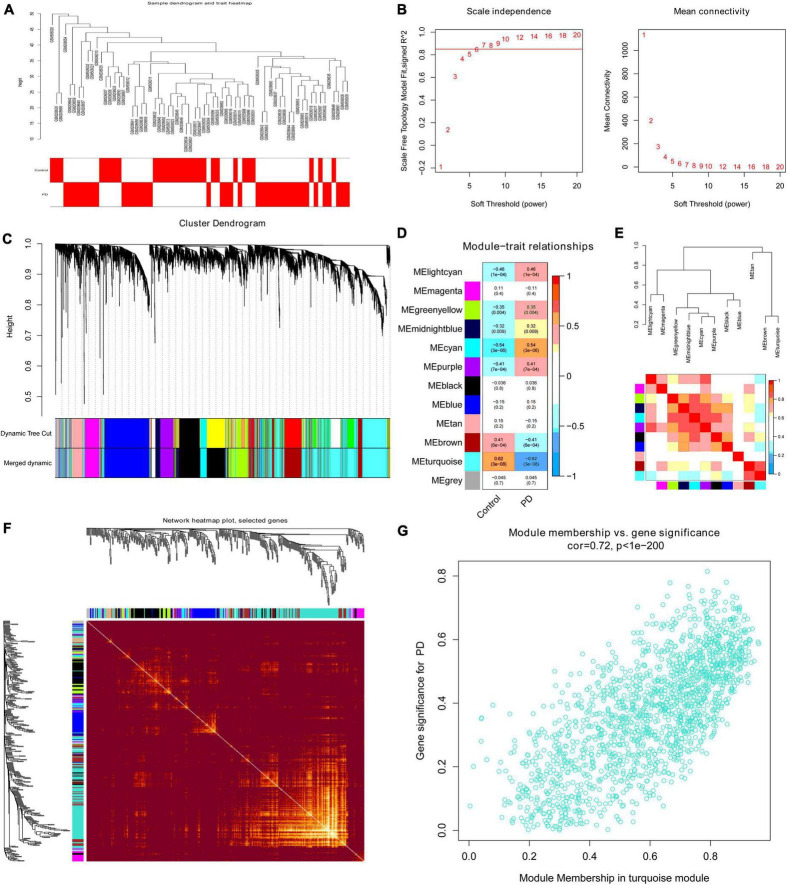
The WGCNA process for the integrated dataset. **(A)** Clustering dendrogram of 38 PD substantia nigra tissue and 29 normal substantia nigra tissue gene expression patterns. **(B)** Analysis of the scale-free fit index (*left*) and the mean connectivity (*right*) for various soft-thresholding powers; the power value β was set as 7 for further analysis. **(C)** Clustering dendrograms of 5000 genes based on a dissimilarity measure (1-TOM). Seventeen co-expression modules were constructed with various colors under the gene tree, and similar modules were merged into twelve modules with a height cutoff of 0.25. Each color represents one module. **(D)** Heatmap of associations between modules and clinical traits. Correlation coefficients and *p*-values are shown in each cell, which were obtained by the intersection of rows and columns. The turquoise module correlated significantly with PD. **(E)** Visualization of the eigengene dendrogram and eigengene adjacency heatmap. Red indicates more similarity, and blue indicates less similarity. **(F)** Visualization of 400 random genes from the WGCNA network using a heatmap plot to depict the TOM among all modules included in the analysis. A redder background indicates a higher module correlation. **(G)** Scatter plot of module membership vs. gene significance for PD in the turquoise module. WGCNA, weighted gene co-expression network analysis; TOM, topological overlap matrix; PD, Parkinson’s disease.

The variance of all genes in integrated dataset was calculated, and the top 5000 variant genes were selected for analysis using the “WGCNA” package. We performed clustered hierarchically analysis of all samples to remove outliers by setting the threshold value to 50, and no outlier samples were removed (Figure 4A). The power of β = 7 (scale-free *R*^2^ = 0.885) was selected as the soft threshold to ensure a scalefree network (Figure 4B). As shown in Figure 4C, 12 modules were finally identified after merging similar modules in the cluster tree by setting the threshold to 0.25. The correlation between module eigengene (ME) values and clinical features is presented in Figure 4D. Seven modules exhibited significant correlation with PD (*p* < 0.05), and the turquoise module represented the highest negative correlation with PD compared (*r* = 0.62; *p* = 3E-8). The correlation between different modules was illustrated through the cluster diagram and heatmap (Figure 4E). Moreover, 400 genes were randomly selected in *R* software to draw a heatmap of the weighted gene co-expression correlations to further illustrate the correlation between different modules (Figure 4F). GS and MM of all modules were calculated to draw scatterplots. As expected, a significant correlation existed in the turquoise module MM and GS (| cor| = 0.72, *p* < 1E-200, Figure 4G), including 1593 genes, which were most significantly associated with PD and selected for further analysis.

### PPI network and candidate hub genes

**FIGURE 5:**
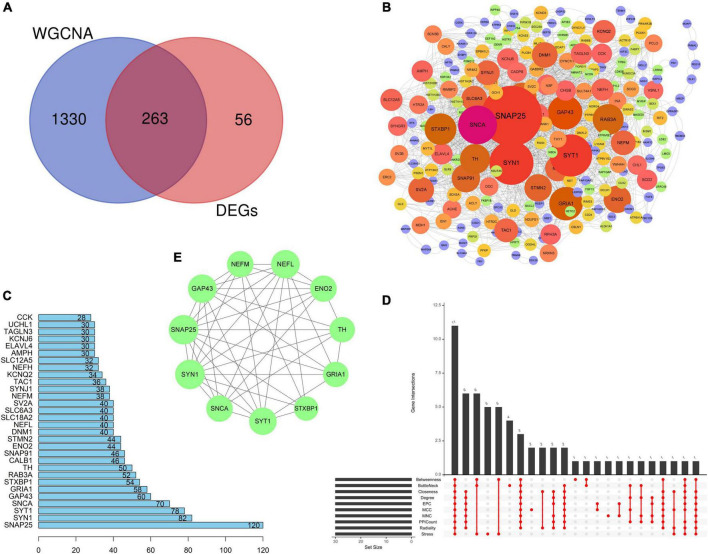
Protein–protein interaction network and identification of candidate hub genes. **(A)** Venn plot showing the intersection between the DEGs and genes in the turquoise module, and 263 CGs were obtained. **(B)** PPI network of CMs by Cytoscape. The size and gradient color of circles are adjusted by the degree value, which reflects the connectivity between nodes. The size of circles has a positive correlation with the degree value. **(C)** PPI Count, revealing the number of adjacent nodes of the top 30 genes (ranked from low to high) based on the PPI network. **(D)** UpSet plot showing the intersection of ten algorithms, namely, MCC (top 30), MNC (top 30), Degree (top 30), EPC (top 30), BottleNeck (top 30), Closeness (top 30), Radiality (top 30), Stress (top 30), and Betweenness (top 30), and PPI Count (top 30). **(E)** Eleven candidate hub genes. All these candidate hub genes were found to be downregulated. PPI, protein–protein interaction; CGs, common genes; MCC, maximal clique centrality; MNC, maximum neighborhood component; EPC, edge percolated component.

Venn analysis was performed based on the DEGs screened from the integrated dataset and the genes in turquoise module, and 263 common genes (CGs) genes were found (Figure 5A). The PPI network of 263 CGs was constructed to investigate the relationships of those genes at the protein level and obtain candidate hub genes using Cytoscape according to the STRING database (Figure 5B). The top 30 genes with the largest number of adjacent nodes were screened as PPICount, including SNAP25, SYN1, SYT1, SNCA, GAP43, GRIA1, STXBP1, RAB3A, TH, and CALB1 (Figure 5C and Supplementary Table 3). Then, we applied nine algorithms to calculate the score of each node gene using the CytoHubba plug-ins of Cytoscape and selected the top 30 genes of each algorithm (Supplementary Table 3). Finally, we screened 11 intersected genes (SNAP25, SNCA, SYT1, ENO2, GRIA, STXBP1, SYN1, TH, NEFM, GAP43, and NEFL) through 10 approaches by “UpSet” in the *R* package (Figures 5D, E). All these intersected genes that were defined as candidate hub genes were found to be downregulated.

### mRNA expression of hub genes in patients

**FIGURE 6:**
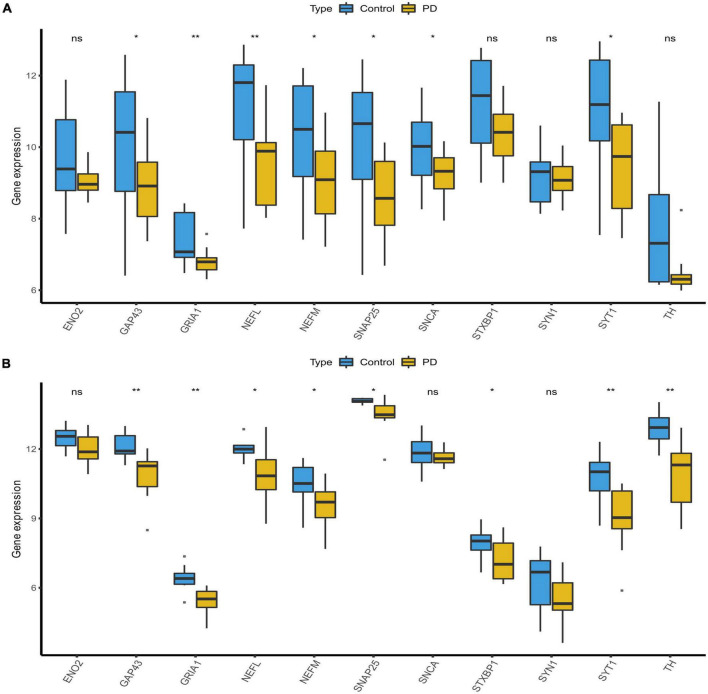
Validation of candidate hub genes. **(A)** Validation of candidate hub genes in the GSE20292 dataset. **(B)** Validation of candidate hub genes in the GSE20681 dataset (*p < 0.05, **p < 0.01). PD, Parkinson’s disease.

The mRNA expression results for the candidate hub genes in the GSE20292 indicated SNAP25, SNCA, SYT1, GRIA, NEFM, GAP43, and NEFL to be expressed at significantly lower levels in the PD group than the control group (Figure 6A, *p* < 0.05). No significant differences were found in mRNA expression of ENO2, STXBP1, SYN1, and TH. In addition, we verified expression of marker genes in the GSE26927 dataset, and SNAP25, SYT1, GRIA, NEFM, GAP43, NEFL, and TH expression was significantly lower in PD patients than normal samples (Figure 6B, *p* < 0.05). Finally, SNAP25, SYT1, GRIA, NEFM, GAP43, and NEFL, which could effectively differentiate PD patients from controls (*p* < 0.05), were selected and considered as the hub genes and potential biomarkers for PD.

### Diagnostic effectiveness of hub genes for PD

**FIGURE 7:**
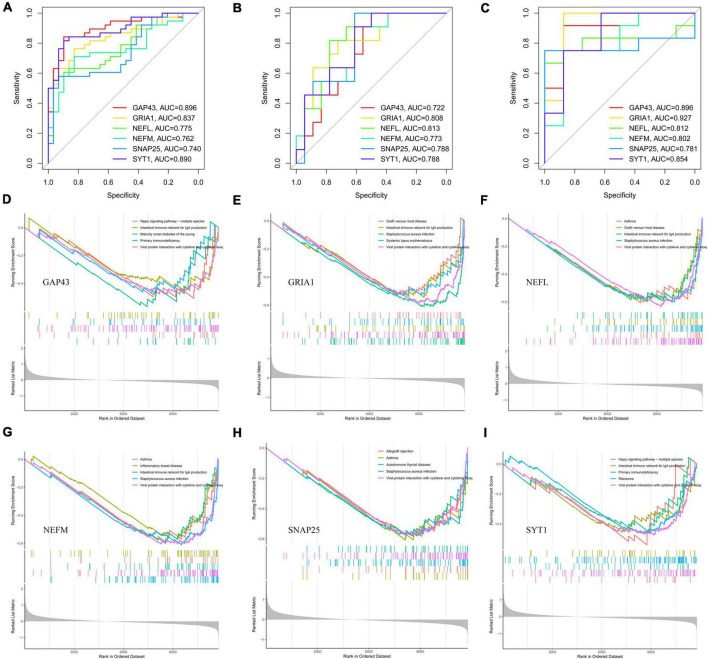
Diagnostic efficacy of potential biomarkers (GAP43, GRIA1, NEFL, NEFM, SNAP25, and SYT1) for prediction of PD and GSEA based on expression levels of those potential biomarkers. ROC analysis of six hub genes (GAP43, GRIA1, NEFL, NEFM, SNAP25, and SYT1) for diagnosing PD in the integrated dataset **(A)** and the validation sets GSE20292 **(B)** and GSE26927 **(C)**. Single-gene GSEA-KEGG pathway analysis of GAP43 **(D)**, GRIA1 **(E)**, NEFL **(F)**, NEFM **(G)**, SNAP25 **(H)**, and SYT1 **(I)**. PD, Parkinson’s disease; GSEA, gene set enrichment analysis; ROC, receiver operator characteristic.

Receiver operating characteristic curves analyses were used to examine the accuracy of the six potential biomarker genes to diagnose PD, with AUC values of 0.896 (GAP43), 0.837 (GRIA1), 0.775 (NEFL), 0.762 (NEFM), 0.740 (SNAP25), and 0.890 (SYT1), respectively, in the training set (Figure 7A). As shown in Figure 7B, the AUC values of GAP43, GRIA1, NEFL, NEFM, SNAP25, and SYT1 were 0.722, 0.808, 0.813, 0.773, 0.788, and 0.788, respectively, in the validation set (GSE20292). Figure 7C indicates that the AUC for all genes was greater than 0.7 in the GSE26927 dataset. The above evidence suggests that GAP43, GRIA1, NEFL, NEFM, SNAP25, and SYT1 can be used as diagnostic biomarkers for differentiating PD patients from normal controls.

### Single-gene GSEA

Parkinson’s disease substantia nigra tissues were divided into two subgroups based on the median expression of the six hub genes. Then, we utilized single-gene GSEA to explore potential signaling pathways of the potential biomarker genes. The top five pathways enriched for potential biomarker genes are illustrated in Figures 7D–I. After comprehensive analysis, we found low NEFM expression to be associated with immune responses (B-cell receptor signaling pathway, Th1 and Th2 cell differentiation, etc.) and various immunologic disease pathways (systemic lupus erythematosus, intestinal immune network for IgA production, etc.), and the neuroinflammation response (NF-kappa B signaling pathway, IL-17 signaling pathway, etc.). The remaining five hub genes are also involved in several immune response pathways or various immunologic disease pathways, including viral protein interaction with cytokine and cytokine receptor (GAP43, GRIA1, NEFL, SNAP25, and SYT1), primary immunodeficiency (GAP43, GRIA1, NEFL, SNAP25, and SYT1), autoimmune thyroid disease (GRIA1, NEFL, SNAP25, and SYT1), and maturity-onset diabetes of the young (GAP43, NEFL, and SNAP25), among others. The details of the KEGG pathway results for the six hub genes are shown in Supplementary Table 5. The above results suggest that these potential marker genes may influence PD development through immune-related pathways.

### ssGSEA of immune infiltration

**FIGURE 8:**
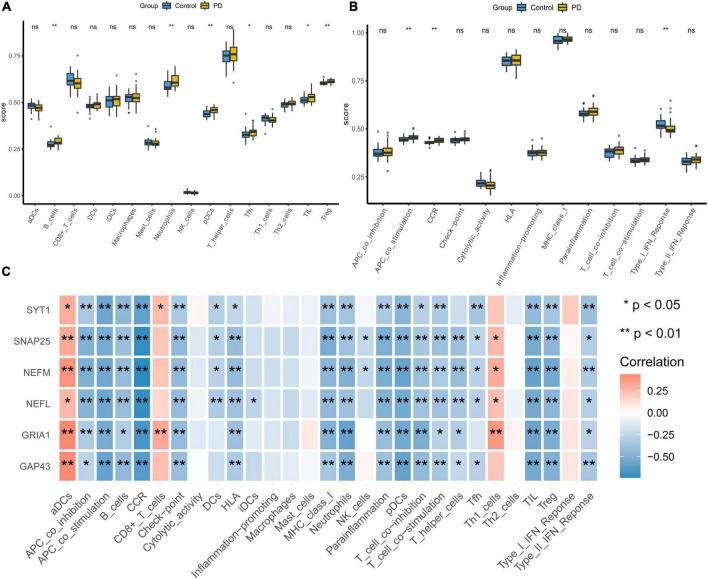
Visualization and evaluation of immune infiltration levels based on ssGSEA. **(A)** Comparison of 16 immune cells between PD samples and control samples. **(B)** Box diagram of different immune function expression levels in the PD and control groups. **(C)** Pearson correlation analysis of 29 types of immune cells and immune-related functions with hub genes (*p < 0.05 and **p < 0.01). PD, Parkinson’s disease.

We evaluated the samples in the integrated dataset by ssGSEA to quantify the immune infiltration and enrichment scores of 29 immune cells and immune-related functions. A heatmap was drawn to investigate correlations between substantia nigra tissue samples with or without PD and immune cells (Supplementary Figure 1A). In the PD group, immune cells such as B cells, neutrophils, plasmacytoid dendritic cells (pDCs), T follicular helper cells (Tfhs), tumor-infiltrating lymphocytes (TILs), and regulatory T cells (Tregs) had higher ssGSEA scores (*p* < 0:05, Figure 8A). Moreover, the box plot illustrated that immune pathways such as APC_co_stimulation and CCR were associated with elevated ssGSEA scores in the PD group, whereas Type_I_IFN_Reponse immune function was lower in the PD group (*p* < 0:05, Figure 8B). The corHeatmap of immune-related functions result showed that check-points were positively related with T_cell_co-stimulation and T_cell_co-inhabition (*r* = 0.84 and 078, respectively, Supplementary Figure 1B). Similarly, parainflammation had a significant positive correlation with CCR (*r* = 0.78, Supplementary Figure 1B). Immune cells such as neutrophils, pDCs, T_helper_cells were positively related with TILs (*r* = 0.77, 0.73, and 0.73, respectively, Supplementary Figure 1C), whereas aDCs were negatively related to Tregs (*r* = −0.42, Supplementary Figure 1C). Correlation analysis showed that most of immune cells had a negative correlation with all of the hub genes (Figure 8C and Supplementary Table 4).

### CIBERSORT analysis of immune infiltration

**FIGURE 9:**
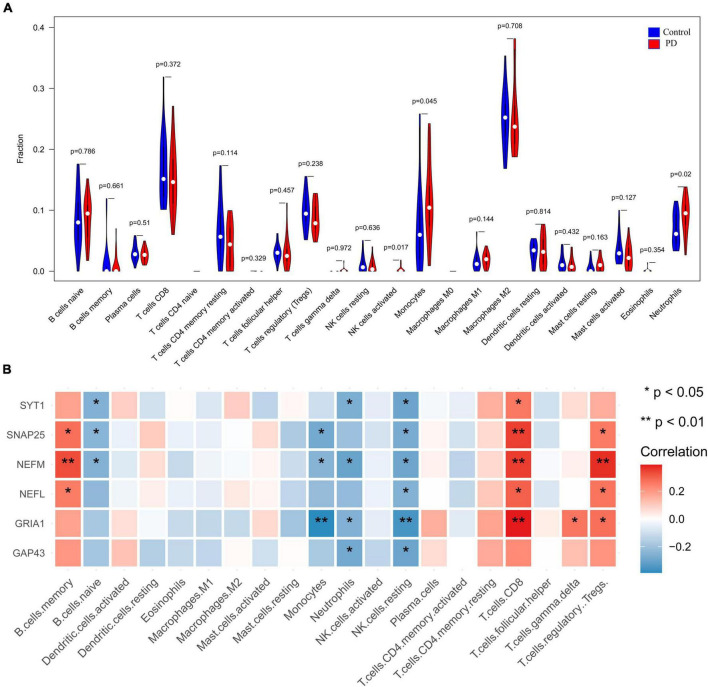
Visualization and evaluation of immune infiltration levels based on the CIBERSORT algorithm. **(A)** Comparison of 22 immune cells between PD samples and control samples. **(B)** Pearson correlation analysis of immune cell infiltration with hub genes (*p < 0.05 and **p < 0.01). PD, Parkinson’s disease.

We performed CIBERSORT analysis to assess infiltrating levels of 22 immune cells in PD samples and normal samples by *R* software. The heatmap showed the relationship between immune cells and all of the samples filtered (Supplementary Figure 2A). The correlation heatmap of 22 types of immune cells demonstrated that T cells regulatory were positively related with T cells CD8 (*r* = 0.56, Supplementary Figure 2B) but that activated dendritic cells and M1 macrophages had a negative correlation (*r* = −0.70); activated mast cells were negatively related to resting mast cells (*r* = −0.69). The violin plot of the immune cells showed that PD patients had a higher level of neutrophils, activated NK cells and monocytes (*p* < 0:05, Figure 9A). Supplementary Figure 2C illustrates the proportion of each type of immune cell in each sample. Pearson correlation analysis revealed a negative correlation for neutrophils with four downregulated genes, including NEFM, GRIA1, SYT1, and GAP43 (Figure 9B and Supplementary Table 4). Combined with above two methods, four genes (NEFM, GRIA1, NEFL, and SYT1) were strongly negatively related to immune infiltration, especially neutrophils, and regarded as marker hub genes related to immune infiltration.

### Construction of a nomogram model to predict occurrence of PD

**FIGURE 10:**
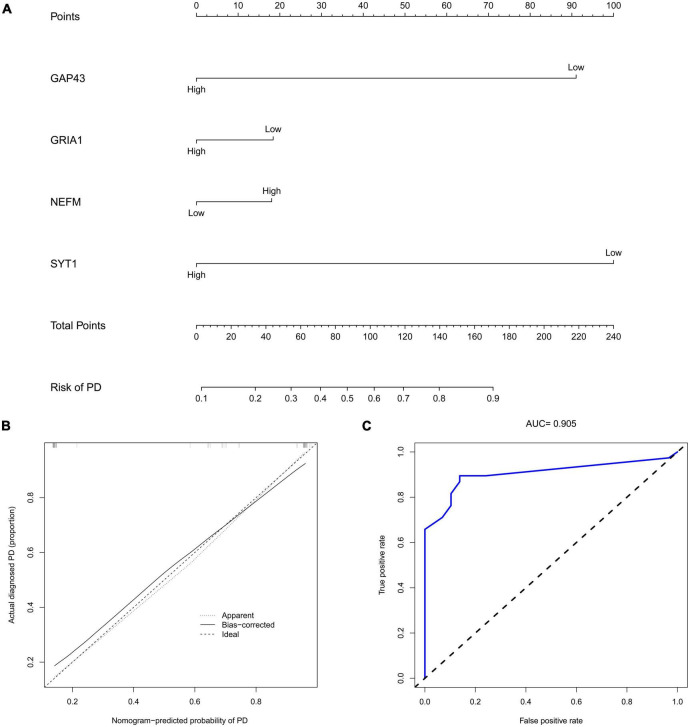
Construction of the nomogram. **(A)** Construction of a nomogram for immune-related hub genes (GAP43, GRIA1, NEFM, and SYT1) for predicting the occurrence of PD. **(B)** Calibration curve estimates the prediction accuracy of the nomogram for PD patients. **(C)** The area under the curve (*AUC*) was 0.905. PD, Parkinson’s disease; AUC, area under the curve.

The marker hub genes, namely, NEFM, GRIA1, NEFL, and SYT1, were then used to construct a nomogram model to predict PD occurrence (Figure 10A). We also utilized calibration plots to confirm the performance of this nomogram model, with a sufficient degree of fit for predicting the incidence of PD (Figure 10B). The AUC was 0.905, suggesting that the predictive model had high predictive accuracy (Figure 10C).

### MiRNA–TF–mRNA regulatory network analysis based on marker genes

**FIGURE 11:**
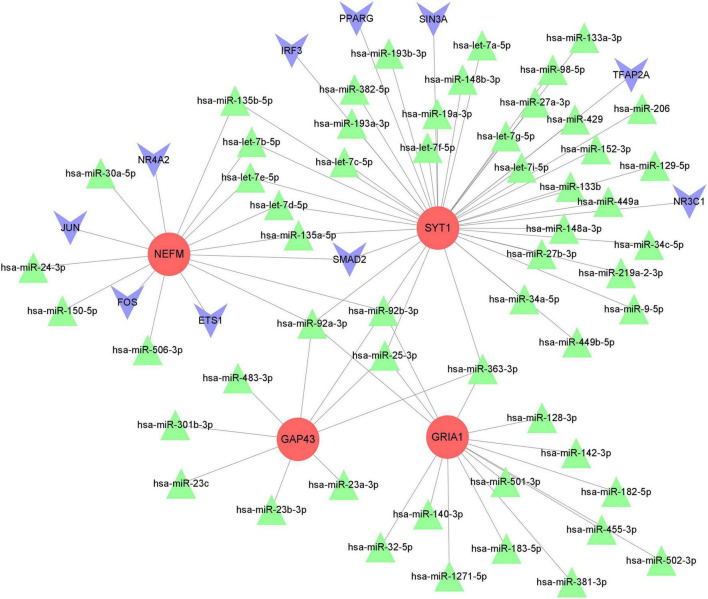
The miRNA-TF-mRNA interaction network. The red circles represent marker hub genes, the blue triangles represent miRNAs, and the green inverted cones indicate TFs. mRNAs, messenger RNAs; miRNAs, microRNAs; TFs, transcription factors.

After miRNA and TF pairs were predicted based on the four marker hub genes, we constructed a miRNA-TF–mRNA network, including 70 miRNAs common in three databases (miRWalk, RNAInter and TargetScan database), 10 TFs, and 4 downregulated marker genes. The regulatory network included 69 nodes and 81 edges, as established through Cytoscape 3.9.1 (Figure 11). Within the network, expression of all marker genes is regulated by hsa-miR-92a-3p, hsa-miR-92b-3p, and hsa-miR-25-3p.

## Discussion

Parkinson’s disease is the most common movement disorder, though its mechanisms have not been fully clarified. This present study used bioinformatics analysis to identify immune infiltration-related marker genes, which were used to construct a nomogram model for the early prediction of PD and miRNA-TF-mRNA network analysis to explore potential therapeutic targets for PD. We obtained a total of 319 DEGs based on the integrated dataset. WGCNA was performed and confirmed the turquoise module as the key module correlating with PD. Intersecting DEGs and genes in the turquoise module were obtained; a total of 263 CMs were screened, which were then used for PPI network construction. We then used 10 approaches based on Cytoscape software and obtained 11 candidate hub genes. Moreover, those 11 genes were validated in the GSE20292 and GSE26927 datasets, with 6 genes, namely, SNAP25, SYT1, GRIA, NEFM, GAP43, and NEFL, screened. ROC analysis demonstrated the effective diagnostic of those six hub genes in PD, suggesting potential for distinguishing PD patients from normal controls.

Six hub genes were selected for further single-gene GSEA by dividing the PD samples into two subgroups based on median expression of the six hub genes. We found that low expression of hub genes was mainly related to the immune response and immune diseases. Then, we analyzed differences in immune cell infiltration level between the substantia nigra of PD samples and healthy brain tissue. Our result indicates that the proportion of neutrophils, monocytes, and activated NK cells were significant higher in PD samples than in controls by CIBERSORT analysis. The ssGAEA algorithm also showed that B cells, neutrophils, pDCs, Tfhs, TILs, Treg, APC_co_stimulation, and CCRs had higher ssGSEA scores in the PD group but that immune function of Type_I_IFN_Reponse was lower in the PD group. The above results revealed that the immune cells (DC, NK cells, T cells, CCRs, neutrophils, and monocytes) play a vital role in the pathogenesis of PD.

Overexpressed α-synuclein induces infiltration of pro-inflammatory monocytes through C-C chemokine receptor type 2 (CCR2) in the CNS, whereas deletion of CCR2 prevents this and subsequent dopaminergic neuronal death in the progression of PD (Harms et al., 2018). Dysregulation of peripheral human monocytes in PD has also been observed (Grozdanov et al., 2014), subsequently inducing a higher infiltration level of monocytes in the cerebrospinal fluid than in the control group (Schröder et al., 2018). The correlation of circulating monocytes and immune cells may be that chemokines lead to increased blood–brain barrier permeability, invasion of peripheral monocytes into the CNS, and infiltration of immune cells (Harms et al., 2018). However, the potential role monocytes in the pathogenesis of PD in humans has not yet been investigated, and further research is warranted.

Increasing evidence suggests that significantly increased T cells (CD8 and CD4) are present in the substantia nigra of PD patients compared with control subjects (Theodore et al., 2008; Harms et al., 2017), and T-cell responses were connected with dopaminergic neuron cell loss (Brochard et al., 2008; Lira et al., 2011; Williams et al., 2021). A growing body of evidence shows that Treg cells, Foxp3-expressing CD4+ CD25+ T lymphocytes, play an important role in immune regulation (Noack and Miossec, 2014). Treg cells protect neurons by inhibiting microglial oxidative stress and inflammation in the central nervous system (CNS) (Reynolds et al., 2007). Huang et al. (2017) revealed that dopaminergic neuronal protection of Treg cells is achieved *via* interaction between CD47 and signal regulatory protein α (SIRPA) in PD processes. Moreover, dysfunction of Treg cells decreases the ability to suppress the function of effector T cells in PD, which may accelerate its progression (Saunders et al., 2012).

T follicular helper cells, which are essential in B-cell activation, are a specialized subtype of CD4+ T cells that are expressed at significantly higher levels in PD patients than in controls (Zhao et al., 2020). In addition, Tfh cells promote Th-17-induced neuroinflammation by inducing inflammatory B-cell responses in the CNS and increasing disease severity (Quinn et al., 2018). Such progression of disease severity can be reduced by inhibiting Tfh cells in the CNS.

Combining two methods, ssGSEA and CIBERSORT, we found that neutrophils were differentially expressed between PD samples and normal samples. The PD-relevant immune response is related not only to changes in brain immune cells and neuroinflammation but also to changes in the peripheral blood system. A recent study reported higher neutrophil counts in PD compared to controls and a decreased lymphocyte count (Jensen and Jacobs, 2021). The neutrophil-to-lymphocyte ratio, which has proven prognostic value in infection, inflammatory diseases and several types of cancers, is significantly higher in PD patients than the peripheral immune profile (Akıl et al., 2015; Muñoz-Delgado et al., 2021). Such an elevated neutrophil population leads to mitochondrial changes, increased markers of oxidative stress, and overexpression of nitric oxide, suggesting that neutrophils participate in the pathological progression of PD (Vitte et al., 2004).

Plasmacytoid dendritic cells can regulate the immune response by producing large amounts of cytokines, particularly type I interferons, which induce B cells to differentiate into plasma cells and produce immunoglobulin (Jego et al., 2003; Poeck et al., 2004; Menon et al., 2016), activate NK-cell cytolytic activity (Colonna et al., 2004), and affect T-cell functions (Agnello et al., 2003). The former is supported by studies in other disease, whereas a direct influence of pDCs in PD remains to be confirmed.

Similarly, Alzheimer’s disease (AD), the most common neurodegenerative disease, is also closely related to immune infiltration (Hu and Wang, 2021; Qian et al., 2022; Zhang et al., 2022). A previous study revealed that some specific immune cells in brain tissue, including Treg cells, activated NK cells, and neutrophils, were significantly more or less abundant in patients with AD than in healthy controls (Hu and Wang, 2021), which is consistent with the results of this study. The infiltration levels of some immune cells, such as pDCs, macrophages, and basophils, were altered in the brain tissue samples of PD and AD patients compared to healthy controls (Qian et al., 2022; Zhang et al., 2022). The potential reasons for this were that the brain tissue samples were derived from different regions and that the pathological mechanisms of these two diseases are not completely the same. The roles of immune infiltration in neurodegenerative diseases still require further investigation.

Moreover, we identified the relationship between the six genes (NEFM, GRIA1, NEFL, SYT1, NEFL, and SNAP25) and immune cell type by CIBERSORT and ssGSEA, and NEFM, GRIA1, NEFL, and SYT1 were found to be closely related to immune cells. KEGG analysis results showed DEGs to be enriched in the synaptic vesicle cycle. Disorders of the synaptic vesicle cycle participate in the pathogenesis of PD and play a critical role in degeneration of dopaminergic neurons. Synaptotagmin-1 (SYT1), a potential target in treating nervous system disorders, regulates neuron exocytosis and the synaptic vesicle cycle (Mingazov and Ugrumov, 2016; Liu and Kaeser, 2019). It has been demonstrated that by sponging miR-34-5p, overexpression of SYT1 has a neuroprotective effect in a mouse model of PD (Shen et al., 2021).

Growth-associated protein-43 (GAP-43), also known as neuromodulin, a marker of synaptic formation and neuronal elongation, plays an essential role in the early stage of nervous system development. PD patients have significantly lower expression levels of GAP-43 in dopaminergic neurons than age-matched controls, which results in reduced regenerative capacity in dopaminergic neurons, as well as involvement of GAP43 downregulation in glial PD pathophysiology (Saal et al., 2017; Chung et al., 2020). Another study revealed that an enriched environment promotes GAP-43 upregulation to induce plastic brain changes and prevent dopaminergic cell loss on the progression of neuronal impairment related to PD (Yuan et al., 2018).

Emerging evidence supports that GluA1 (also known as GRIA1), a subunit of a-amino-3-hydroxy-5-methyl-4-isoxazolepropionic acid (AMPA) receptors, mediates synaptic plasticity, thereby playing a critical role in brain function and dysfunction (Qu et al., 2021). The GluA1-homomeric form, a calcium-permeable AMPA receptor subtype, induces trafficking and insertion of AMPARs (AMPA receptors) in synapses (Zhang and Abdullah, 2013; Tamano et al., 2018). Defects in regulated AMPAR trafficking can lead to movement disorders, which may be involved in the pathogenesis of PD (Tamano et al., 2018). One study demonstrated that the mechanism for the treatment of Alzheimer’s disease (Qu et al., 2021) is mainly related to upregulation and phosphorylation of GluA1.

Neurofilament proteins, composed of a triplet of neurofilament medium chain (NFM), heavy chain (NFH), and light chain (NFL) proteins according to their molecular weight, are commonly used as reliable biomarkers for neurodegenerative pathology (Zucchi et al., 2020). It has been demonstrated that NEM, also known as NEFM, is linked to regulatory functions in dopaminergic neurotransmission (Kim et al., 2002) and is associated with the immune response (Barboni et al., 2014; Li et al., 2021). Increased levels of NEFM have been detected in various neurological diseases, such as brain damage (Martínez-Morillo et al., 2015), schizophrenia spectrum disorders (Runge et al., 2022), and amyotrophic lateral sclerosis (Häggmark et al., 2014). However, the association of NEFM with PD has not been reported previously and requires further investigation.

We also investigated the associations between four biomarker genes and AD. NEFM (Mirza and Rajeh, 2017; Hu et al., 2020) and SYT1 (Mirza and Rajeh, 2017) were notably downregulated in AD compared with control brain tissues in previous studies, demonstrating that these two genes are linked to the pathogenesis of AD. In a postmortem study, the hippocampal expression levels of GRIA1, GAP-43, and NEFM were significantly decreased in AD patients compared with controls (Chowdhury et al., 2020). In addition, the CSF levels of both GAP-43 and SYT1 significantly increased in patients with dementia due to AD, implying that these genes can potentially be used as biomarkers of synaptic dysfunction to predict the progression of AD (Öhrfelt et al., 2016; Qiang et al., 2022). The above evidence identifies four biomarker genes associated with AD; however, their roles in this disease still require further research.

Various biomarkers for early PD diagnosis have been proposed (Surguchov, 2022), but they remain investigational and need further confirmation. Recent studies have demonstrated that exosomes mediate the transfer of α-synuclein protein to brain cells, providing a potential mechanism for the propagation of pathological α-synuclein aggregation in brain cells and the acceleration of pathology in PD (Pinnell et al., 2021; Valencia et al., 2022). α-Synuclein aggregation in CSF as detected by protein misfolding cyclic amplification (PMCA) and real-time quaking-induced conversion (RT-QuIC) had high diagnostic accuracy (AUC 0.93 and 0.89, respectively) in distinguishing PD patients from controls (Kang et al., 2019). Pathological α-synuclein in plasma neuron-derived exosomes can serve as a biomarker to differentiate PD patients from healthy controls (Kluge et al., 2021). Moreover, CNS-derived exosomes in plasma were significantly higher in PD patients than in controls, whereas the performance of plasma exosomal α-synuclein was only moderate (AUC 0.654). We should conduct further research to shed more light on this extraordinary phenomenon. Whether the four biomarker genes drive this discrepancy by interacting with or regulating α-synuclein aggregation in fluid or neuron-derived exosomes and lead to this discrepancy also remains to be explored.

Next, the four immune-related biomarkers were selected as key genes for further miRNA-TF-mRNA network analysis. Taking the miRNA-TF-mRNA network into account, we explored the regulatory mechanisms of NEFM, GRIA1, NEFL, and SYT1, which might be regulated by hsa-miR-92a-3p and hsa-miR-92b-3p. Our findings indicate that hsa-miR-92a is the hub miRNA in both regulatory and co-expression networks and has a strong functional role in PD. Based on integrated network analysis, hsa-miR-25-3p and hsa-miR-363-3p were also identified as bridges connecting to GRIA1, NEFL, and SYT1. Campos-Melo et al. demonstrated that miR-92a-3p is expressed in motor neurons of the spinal cord and can directly downregulate NEFM. MiR-92a is also able to repress translation of GluA1 receptors to block homeostatic scaling in rats (Letellier et al., 2014). Moreover, researchers have found that an inhibitor of miR-363 increases the expression level of GAP43 in glioma cells (Conti et al., 2016). Interestingly, miR-25, miR-92a-3p (miR-92a-1 and miR-92a-2), and miR-363 all belong to the miR-92 family, a group of highly conserved miRNAs (Olive et al., 2010). MiR-92a may be viewed as a potential therapeutic target for PD. Previous studies have revealed aberrant expression of miR-92a in various cancers, and it exerts its function in tumors mainly by promoting cell proliferation, invasion and metastasis and inhibiting apoptosis (Dou et al., 2020; Feng et al., 2021). Overexpression of miR-92a suppresses immune cell function in many kinds of malignant tumors (Dou et al., 2020; Feng et al., 2021). However, the regulatory mechanisms of miR92a have rarely been studied in PD. Thus, we speculate that the miR92a family may regulate the identified biomarkers to participate in immune infiltration in PD, and further studies to explore the pathophysiology of the miR92a family in PD are required in the future.

Finally, we developed a nomogram to predict the occurrence in PD patients based on the four immune-related biomarkers. The results showed that the nomogram model had excellent individual predictive effects. Therefore, this nomogram may provide new insight and contribute to accurate diagnosis of PD, particularly for early stage PD.

To the best of our knowledge, this is the first diagnostic nomogram to predict PD based on GEO datasets. However, there are still some limitations in this study. First, although we performed rigorous bioinformatics analysis and external validation to verify expression of the hub genes and their predictive power in PD diagnosis, the results need to be thoroughly investigated in *in vitro* experiments. Second, there are few datasets of miRNA expression in the substantia nigra of PD, and the relationship of miRNAs and immune infiltration should be investigated. Further studies are warranted to focus on miRNAs with mechanisms in PD. Finally, although a nomogram to predict PD is presented, this analysis was based on genome-wide expression of the substantia nigra from postmortem brains, and detection of substantia nigra mRNA expression in practical applications is difficult to implement. Future research should focus on comparing gene expression and immune infiltration patterns between different neurodegenerative diseases, enabling the identification of early stage disease biomarkers that can improve the understanding of the pathophysiology of neurodegenerative diseases and facilitate the application of timely symptomatic interventions. Nevertheless, this study provides new insight into exploring the mechanism of PD and PD diagnosis.

## Conclusion

In conclusion, this study not only suggests that immune cell infiltrates are associated with PD but also presents four effective diagnostic immune-related biomarkers for PD patients. We also predict that the miRNA-92a family might target these immune-related biomarkers in regulating PD. Our research provides further insight into potential therapeutic targets for PD.

## Data availability statement

The original contributions presented in this study are included in the article/Supplementary material, further inquiries can be directed to the corresponding author.

## Author contributions

PZ, YX, and LZ contributed to the design and initiation of the study. PZ, YX, LZ, HoL, and JS collected and analyzed the data. PZ and YX drafted the manuscript. LZ, HoL, JS, and HuL critically revised the content. All authors reviewed and revised the final manuscript.

## Footnotes

1
https://www.ncbi.nlm.nih.gov/geo/

2
https://www.r-project.org/

3
www.string-db.org

4
http://amp.pharm.mssm.edu/Enrichr/
