# Supplementary material for: Novel diagnostic biomarkers related to immune infiltration in Parkinson’s disease by bioinformatics analysis
Source: Front Neurosci. 2023 Jan 26;17:1083928. doi: 10.3389/fnins.2023.1083928 (PMC9909419; doi:10.3389/fnins.2023.1083928)
Supplement: Supplementary file 1 [file Data_Sheet_1.docx]

Supplementary Material

Novel diagnostic biomarkers related to immune infiltration in Parkinson’s disease by bioinformatics analysis


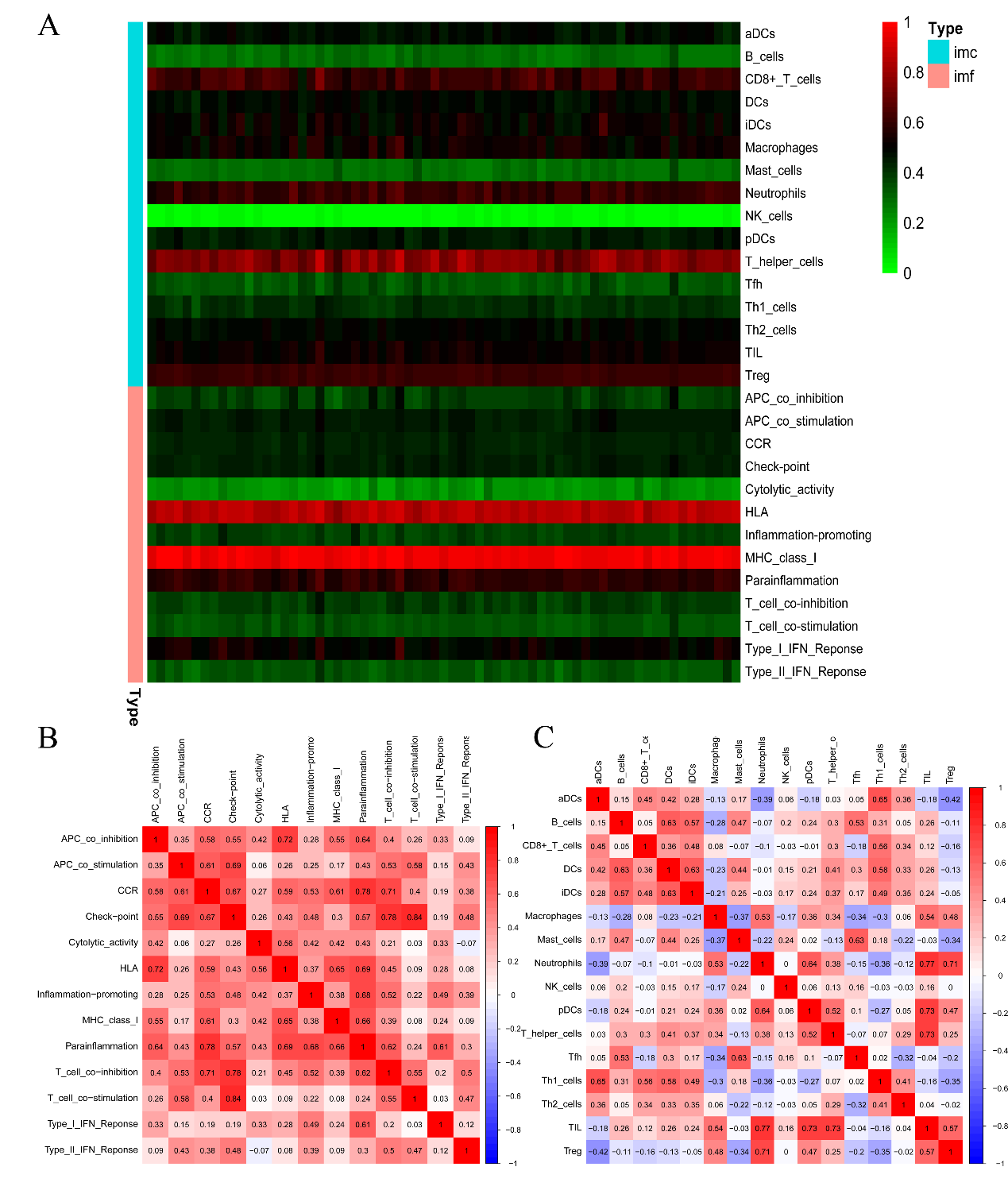


**Supplementary Figure1** Visualization and evaluation of immune infiltration levels based on ssGSEA. (A) Heat map of 29 types of immune cells and immune-related functions in control patients and PD patients. Correlation matrix of infiltration degree of 13 immune functions (B), and 16 immune cells (E). Red indicates trends consistent with the positive correlation and blue means negative correlation between two immune functions cells.


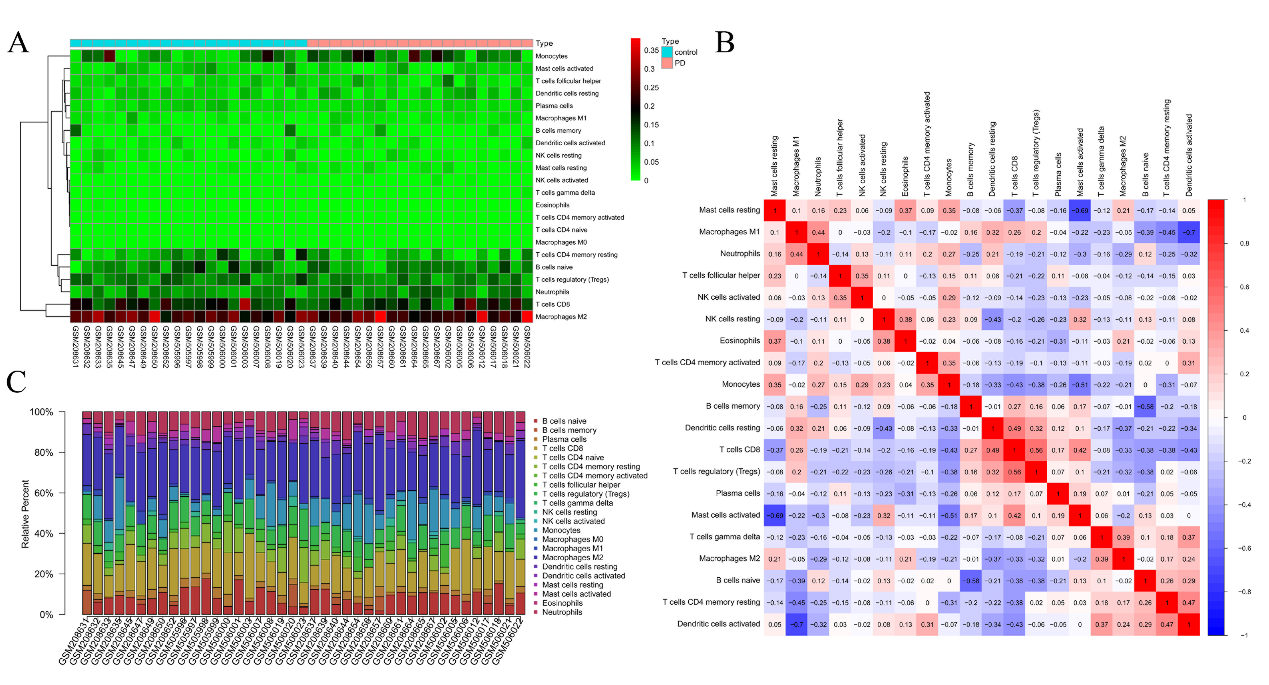


**Supplementary Figure2** Visualization and evaluation of immune infiltration levels based on CIBERSORT algorithm. (A) Heat map of 22 types of immune cells in normal patient and PD patients. (B) Correlation matrix of infiltration degree of 20 type immune cells. Red indicates trends consistent with the positive correlation and blue means negative correlation between two immune cells. (C) Stacked bar chart of 22 kinds of immune cells.
