# Supplementary material for: Novel diagnostic biomarkers related to immune infiltration in Parkinson’s disease by bioinformatics analysis
Source: Front Neurosci. 2023 Jan 26;17:1083928. doi: 10.3389/fnins.2023.1083928 (PMC9909419; doi:10.3389/fnins.2023.1083928)
Supplement: Supplementary file 2 [file Table_1.DOCX]

| **GEO ID** | **Platform** | **Tissue** | **Control** | **PD** | **Experiment**  **type** | **Attribute** |
| --- | --- | --- | --- | --- | --- | --- |
| GSE20163 | GPL96 | Substantia nigra | 9 | 8 | Array | Test |
| GSE20164 | GPL96 | Substantia nigra | 5 | 6 | Array | Test |
| GSE8397 | GPL96 | Substantia nigra | 15 | 24 | Array | Test |
| GSE20162 | GPL96 | Substantia nigra | 18 | 11 | Array | Validation |
| GSE26927 | [GPL6255](https://www.ncbi.nlm.nih.gov/geo/query/acc.cgi?acc=GPL6255) | Substantia nigra | 8 | 12 | Array | Validation |

**Supplementary Table S1** Detailed information for the 5 GEO datasets in this study.

Abbreviations: GEO, gene expression omnibus; GSE, GEO series; GPL, GEO platform; PD, parkinson's disease.
