# Supplementary material for: Novel diagnostic biomarkers related to immune infiltration in Parkinson’s disease by bioinformatics analysis
Source: Front Neurosci. 2023 Jan 26;17:1083928. doi: 10.3389/fnins.2023.1083928 (PMC9909419; doi:10.3389/fnins.2023.1083928)
Supplement: Supplementary file 4 [file Table_3.DOCX]

**Supplementary Table S3. Candidate** hub genes identified using the 10 algorithms

| **Rank** | **9 methods in the CytoHubba plugin** | | | | | | | | | **PPIcount** |
| --- | --- | --- | --- | --- | --- | --- | --- | --- | --- | --- |
|  | **Betweenness** | **BottleNeck** | **Closeness** | **Degree** | **EPC** | **MCC** | **MNC** | **Radiality** | **Stress** |  |
| 1 | SNAP25 | SNCA | SNAP25 | SNAP25 | SNAP25 | SNAP25 | SNAP25 | SNAP25 | MAGED1 | SNAP25 |
| 2 | SNCA | SNAP25 | SYN1 | SYN1 | SYN1 | SYN1 | SYN1 | SYN1 | NREP | SYN1 |
| 3 | SYT1 | STXBP1 | SYT1 | SYT1 | SYT1 | SYT1 | SYT1 | SYT1 | DLK1 | SYT1 |
| 4 | ENO2 | ENO2 | SNCA | SNCA | SNCA | GAP43 | SNCA | SNCA | SNAP25 | SNCA |
| 5 | GRIA1 | YWHAH | GAP43 | GAP43 | GAP43 | SNCA | GAP43 | GAP43 | PEG10 | GAP43 |
| 6 | STXBP1 | ITPR1 | STXBP1 | GRIA1 | TH | STXBP1 | GRIA1 | STXBP1 | TUBB2A | GRIA1 |
| 7 | SYNJ1 | RAB3A | RAB3A | STXBP1 | CALB1 | TH | STXBP1 | RAB3A | PNMAL1 | STXBP1 |
| 8 | RAB3A | TAGLN3 | GRIA1 | RAB3A | STXBP1 | ENO2 | TH | TH | RET | RAB3A |
| 9 | SYN1 | NSF | TH | TH | GRIA1 | SNAP91 | RAB3A | NEFL | STXBP1 | TH |
| 10 | KCNQ2 | TUBB2A | NEFL | CALB1 | SV2A | SV2A | CALB1 | GRIA1 | TH | CALB1 |
| 11 | TH | GRIA1 | CALB1 | SNAP91 | NEFL | RAB3A | SNAP91 | CALB1 | SNCA | SNAP91 |
| 12 | YWHAH | SV2A | SNAP91 | STMN2 | SNAP91 | CALB1 | STMN2 | SV2A | SULT4A1 | ENO2 |
| 13 | DIRAS2 | STMN2 | SV2A | ENO2 | RAB3A | NEFH | SLC18A2 | DNM1 | ENO2 | STMN2 |
| 14 | MAGED1 | DIRAS2 | DNM1 | SLC6A3 | SLC18A2 | DNM1 | SV2A | TAC1 | BEX1 | DNM1 |
| 15 | DNM1 | TH | ENO2 | NEFL | ENO2 | TAC1 | SLC6A3 | ENO2 | DIRAS2 | NEFL |
| 16 | NEFM | SYNJ1 | SLC18A2 | SLC18A2 | STMN2 | SYNJ1 | NEFL | SLC18A2 | NEFM | SLC18A2 |
| 17 | GAP43 | DNM1 | SYNJ1 | SV2A | NEFM | SLC18A2 | TAC1 | NEFM | SYT1 | SLC6A3 |
| 18 | TUBB2A | MAGED1 | SLC6A3 | DNM1 | TAC1 | UCHL1 | ENO2 | SNAP91 | KCNQ2 | SV2A |
| 19 | SLC6A3 | NEFL | NEFM | NEFM | NEFH | AMPH | NEFM | SLC6A3 | TBC1D9 | NEFM |
| 20 | NREP | SYN1 | TAC1 | SYNJ1 | SYNJ1 | SYNGR3 | NEFH | SYNJ1 | GRIA1 | SYNJ1 |
| 21 | SULT4A1 | NEFM | STMN2 | TAC1 | UCHL1 | NEFL | SLC12A5 | NEFH | SYN1 | TAC1 |
| 22 | ITPR1 | NEFH | NEFH | KCNQ2 | SLC6A3 | PCLO | DNM1 | AMPH | GAP43 | KCNQ2 |
| 23 | RET | GAP43 | AMPH | NEFH | DNM1 | NEFM | KCNQ2 | STMN2 | LRRC49 | NEFH |
| 24 | NEFL | NREP | KCNQ2 | SLC12A5 | SLC12A5 | GRIA1 | UCHL1 | UCHL1 | CHL1 | SLC12A5 |
| 25 | GCH1 | KCNJ6 | ELAVL4 | TAGLN3 | KCNQ2 | STMN2 | CCK | ELAVL4 | YWHAH | AMPH |
| 26 | NEFH | SYT1 | UCHL1 | UCHL1 | AMPH | ACHE | CHGB | KCNQ2 | PRKAR2B | ELAVL4 |
| 27 | DLK1 | GABRB1 | SLC12A5 | KCNJ6 | ELAVL4 | SLC6A3 | ELAVL4 | INA | GCH1 | KCNJ6 |
| 28 | BEX1 | CADPS | RPH3A | AMPH | CHGB | KCNJ6 | TAGLN3 | CHL1 | GPRASP1 | TAGLN3 |
| 29 | PRKAR2B | HIST1H1C | INA | ELAVL4 | CCK | CHL1 | SCG2 | RPH3A | SYNJ1 | UCHL1 |
| 30 | THY1 | RET | CHL1 | CCK | SYNGR3 | KCNQ2 | AMPH | SLC12A5 | NEFL | CCK |

Abbreviations: PPI, protein-protein interaction; MCC, Maximal Clique Centrality; MNC, Maximum Neighborhood Component; EPC, Edge Percolated Component.
